# Preferential signal pathways during the perception and imagery of familiar scenes: An effective connectivity study

**DOI:** 10.1002/hbm.26313

**Published:** 2023-05-23

**Authors:** Maria Giulia Tullo, Hannes Almgren, Frederik Van de Steen, Maddalena Boccia, Federica Bencivenga, Gaspare Galati

**Affiliations:** ^1^ Department of Translational and Precision Medicine Sapienza University Rome Italy; ^2^ Brain Imaging Laboratory, Department of Psychology Sapienza University Rome Italy; ^3^ Department of Data Analysis, Faculty of Psychology and Educational Sciences Ghent University Ghent Belgium; ^4^ Department of Clinical Neurosciences, Cumming School of Medicine University of Calgary Calgary Alberta Canada; ^5^ Hotchkiss Brain Institute University of Calgary Calgary Alberta Canada; ^6^ AIMS, Center For Neurosciences Vrije Universiteit Brussels Brussels Belgium; ^7^ Department of Psychology La Sapienza University Rome Italy; ^8^ Cognitive and Motor Rehabilitation and Neuroimaging Unit Santa Lucia Foundation (IRCCS Fondazione Santa Lucia) Rome Italy; ^9^ PhD program in Behavioral Neuroscience Sapienza University of Rome Rome Italy

**Keywords:** dynamic causal modeling, fMRI, navigation, resting‐state functional connectivity, visual imagery, visual perception

## Abstract

The perception and imagery of landmarks activate similar content‐dependent brain areas, including occipital and temporo‐medial brain regions. However, how these areas interact during visual perception and imagery of scenes, especially when recollecting their spatial location, remains unknown. Here, we combined functional magnetic resonance imaging (fMRI), resting‐state functional connectivity (rs‐fc), and effective connectivity to assess spontaneous fluctuations and task‐induced modulation of signals among regions entailing scene‐processing, the primary visual area and the hippocampus (HC), responsible for the retrieval of stored information. First, we functionally defined the scene‐selective regions, that is, the occipital place area (OPA), the retrosplenial complex (RSC) and the parahippocampal place area (PPA), by using the face/scene localizer, observing that two portions of the PPA—anterior and posterior PPA—were consistently activated in all subjects. Second, the rs‐fc analysis (*n* = 77) revealed a connectivity pathway similar to the one described in macaques, showing separate connectivity routes linking the anterior PPA with RSC and HC, and the posterior PPA with OPA. Third, we used dynamic causal modelling to evaluate whether the dynamic couplings among these regions differ between perception and imagery of familiar landmarks during a fMRI task (*n* = 16). We found a positive effect of HC on RSC during the retrieval of imagined places and an effect of occipital regions on both RSC and pPPA during the perception of scenes. Overall, we propose that under similar functional architecture at rest, different neural interactions take place between regions in the occipito‐temporal higher‐level visual cortex and the HC, subserving scene perception and imagery.

## Graphical abstract

While recruiting common areas, visual perception and imagery obey different principles. By using effective connectivity we show that the information flow follows a postero‐anterior (i.e., from visual to high‐order areas) and an antero‐posterior progression (i.e., the opposite direction) during the visual perception and the visual imagery of familiar landmarks, respectively.

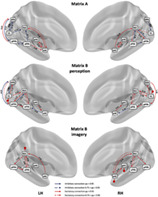

## 1 INTRODUCTION

Human brain regions underlying the perception of scenes and spatial navigation span from lower‐level sensory to higher‐level cognitive areas. The visual information arises in primary visual areas and crosses parieto‐temporal and medial regions, such as the occipital place area (OPA), the parahippocampal place area (PPA), and the retrosplenial complex (RSC), through which a cognitive map of the surrounding environment is gradually built. Specifically, RSC is positioned in between the parietal and the medial lobes in such a way to translate the egocentric spatial code to allocentric spatial codes and vice versa (Burgess, 2006, 2008; Epstein, 2008; Maguire, 2001). Subsequently, regions such as the entorhinal cortex and the hippocampus (HC) build a stable representation of the world (Nau et al., 2020). These spatial representations shape our memory and draw up a mental image of space to be retrieved in the absence of perceptual stimulation.

Neuroimaging and neuropsychological studies pointed out that both perception and imagery share similar cerebral structures that depend on the content of the image to be perceived or imagined (Dijkstra et al., 2017; Ganis et al., 2004; Thorudottir et al., 2020). For instance, perceiving or imagining a face (or a scene) leads to similar activations in face‐ (or scene‐) selective brain regions in the occipito‐temporal high‐level visual cortex (HVC; Boccia et al., 2019; Mechelli, 2004; O'Craven & Kanwisher, 2000). Nonetheless, to date, it remains unclear whether these regions differently communicate with each other during visual perception and imagery and, specifically, during the observation of visuo‐spatial stimuli and the retrieval of the correspondent spatial mental images.

The anatomical and functional neural substrates of the spatial navigation pathway have been accurately studied in non‐human primates (Kravitz et al., 2011): this pathway originates from the primary visual area (V1), which is strongly connected with the middle temporal (MT) area, V2, V3, and V4 visual areas (Felleman & Van Essen, 1991). At the same time, MT is strongly interconnected with the caudal portion of the inferior parietal lobule (cIPL), a hub from which several goal‐directed pathways branch off. The cIPL is indirectly connected with the posterior cingulate cortex (PCC) and the retrosplenial cortex. These regions directly project to presubiculum and parasubiculum hippocampal subdivisions and to the posterior parahippocampal regions TFO and TH/TF (Saleem et al., 2007). Concurrently, parahippocampal areas project to presubiculum and parasubiculum hippocampal subdivisions as well (Insausti et al., 1987; Kravitz et al., 2011; Sewards, 2011).

Human homologs of the primate brain areas active during spatial navigation tasks have been discovered (Margulies et al., 2009). It was demonstrated that the PPA is active more for passive viewing of real‐world scenes than for objects and faces (Epstein & Kanwisher, 1998) showing a preference for the upper visual field (Silson et al., 2015). Located at the boundary between the posterior parahippocampal cortex and the anterior lingual gyrus, PPA is considered the human homolog of the primate area TFO and TH/TF (Aguirre et al., 1998; Vincent et al., 2010). It has been proposed that PPA is composed of at least two functional units that lie on its anterior–posterior axis that may respectively correspond to the macaque TFO and TH/TF (Sewards, 2011). The functional connectivity of the two anterior and posterior units of PPA was investigated by Baldassano et al. (2013) applying a functional connectivity method for examining connectivity differences within regions of interest (ROIs; Baldassano et al., 2012). These authors found that the two PPA portions were differently connected with regions belonging to the navigational pathway: the anterior PPA is more connected with the medial regions such as RSC and HC, specifically the anterior portion. On the contrary, the posterior PPA showed a strong connectivity with occipital regions such as OPA (Baldassano, Esteva, et al., 2016).

Another region that is considered part of the spatial‐navigation system is the RSC, which is composed of the retrosplenial cortex itself, the PCC, and the anterior bank of the parietal‐occipital fissure (Burles et al., 2017). Functional magnetic resonance imaging (fMRI) studies found that together with PPA, RSC responds more to scenes than other types of stimuli, that is, faces and objects and it is crucially involved in navigation since it builds a cognitive map of the surrounding environment (Sewards, 2011). Indeed, the activation of RSC increases whenever subjects localize or orient themselves within the spatial scene (Epstein, 2008; Sulpizio et al., 2013). Moreover, RSC is strongly activated by the imagination of familiar scenes, suggesting that it also has a mnemonic role.

The HC has been also suggested to be crucial for navigational tasks. Since O'Keefe and Nadel (1978) found that the HC has a fundamental role in spatial learning, several studies investigated its contribution in navigational tasks (Rodriguez, 2010; Vass & Epstein, 2013). Among others, it has been proposed that the HC is essential in navigation since it encodes and consolidates the flexible expression of memories (Eichenbaum, 2012, 2017; Eichenbaum & Cohen, 2014).

Finally, the most dorsal region entailing with the perception of the scene is the OPA, also termed as transverse occipital sulcus (TOS; Dilks et al., 2013), which partially overlap the dorsal V3A and V3b, LO1, and LO2 (Bettencourt & Xu, 2013; Nasr et al., 2011; Silson et al., 2016). Silson et al. (2015), studying the retinotopic organization of OPA, found a preference of this region for the lower visual field. Likewise, Julian et al. (2016) proposed that OPA is primarily involved in the processing of the spatial aspects of a scene, such as the environment boundaries. In light of these findings, it was proposed that OPA extracts the spatial features of the scene, like the near navigation affordances, to guide the encoding of multiple potential paths, choosing the most accessible (Bonner & Epstein, 2017).

Although the human functional properties of navigationally relevant regions have been deeply studied, the bi‐directional interactions among these regions during the perception of familiar scenes and the retrieval of the relative mental images are still unknown. In the present study, we first performed resting‐state functional connectivity (rs‐fc) analysis on an fMRI data set of 77 subjects from our database to study the functional connectivity profile at rest of scene‐selective regions, the primary visual area V1, and the HC. Second, we applied dynamic causal modelling (DCM) approach (Friston et al., 2003) by re‐analyzing an fMRI data set from Boccia et al.'s (2017) study, where 16 participants observed or were asked to imagine a set of familiar landmarks. Boccia et al. (2017) showed that the neural interactions between PPA, RSC, and HC were modulated by the task, with the PPA more coupled with RSC during perception and with HC during imagery. Here, we broaden the neural network assigned to scene perception and imagery and, by performing an effective connective analysis, we expected to find evidence of task‐specific couplings linking the above‐mentioned brain areas. Based on previous studies, we hypothesized that perception and imagery would show opposite streams: during perception, we expected connectivity going from low‐ to higher‐level cognitive areas similar to the macaque circuit; during imagery, we expected to see connectivity in the opposite direction because of the reactivation of previously stored mental traces.

## 2 METHODS

### 2.1 Participants

The present study is based on the re‐analysis of BOLD data from two data sets. The first one included fMRI resting‐state data of 77 subjects (35 females, mean age = 30 years, range 24–36 years), some of which (44 subjects) also underwent a functional localizer for scene‐selective regions.

The second data set included BOLD data from 14 healthy subjects (three females, mean age 26 years, range 24–28 years) who participated in a previous study of ours (Boccia et al., 2017) intending to study the neural communication among PPA, RSC, and HC during perception and imagery of familiar places. Here, participants were asked to observe and imagine landmarks they knew very well. Intriguingly, both the imagery and perception tasks required to recollect the spatial location of the landmarks. Boccia et al.'s (2017) study included three scans of a scene perception/imagery experiment (henceforth named “campus experiment”) and two localizer scans for scene‐selective regions.

All the participants had no history of neurological or psychiatric disorders, had normal or corrected‐to‐normal vision, and gave their written informed consent to participate in the study. This study was approved by the local research ethics committee of the IRCCS Fondazione Santa Lucia in Rome, according to the Declaration of Helsinki.

### 2.2 Stimuli

In the resting‐state fMRI experiments, subjects were asked to lie in the MRI scanner with their eyes closed thinking about nothing in particular and no experimental task was imposed, whereas during the localizer experiment subjects lay in the MRI scanner and passively viewed stationary pictures of scenes and faces. The localizer aimed at identifying scene‐responsive regions (i.e., OPA, PPA, and RSC) and each scan consisted of eight alternative blocks (16 s) of passive viewing of indoor (50%) and outdoor (50%) scenes and eight alternative blocks (16 s) of male (50%) and female (50%) face pictures with neutral expressions, presented for 300 ms every 500 ms, interleaved with a fixation period of 15 s on average.

In the campus experiment, participants were asked to recall the spatial position of the landmarks, i.e., buildings within the Sapienza University campus, in both perceptual and imagery trials. Specifically, pictures of the buildings were presented in the perceptual trials, whereas written labels were displayed on the screen in imagery trials (for a visual representation of stimuli and timeline, see fig. 1 in Boccia et al., 2017). A total of eight landmarks were included in the experiment. Participants were asked to recall the spatial position of the landmarks viewed during perceptual trials, to imagine the written landmark from the point of view of the building façade, and to recall its spatial position during imagery trials. Each fMRI scan included six repetitions per item, for a total amount of 48 perceptual and 48 imagery trials. Trials lasted 2 seconds (s) and were interleaved by a fixation cross enduring 2 s.

### 2.3 Image acquisition

Functional T2*‐weighted images of the first and second data sets were collected using a gradient‐echo EPI sequence using Blood‐Oxygenation Level‐Dependent (BOLD) contrast over the whole brain (Kwong et al., 1992) on a 3 T Siemens Allegra scanner (Siemens Medical Systems, Erlangen, Germany) and included 30 contiguous 4 mm slices acquired with an in‐plane resolution of 3 × 3 mm in an interleaved excitation order (echo time [TE] = 30 ms, repetition time [TR] = 2 s, and flip angle = 70°). Head movements were minimized using cushions. Stimuli were generated by a control computer located outside the MR room, running an in‐house software implemented in MATLAB. An LCD video projector projected stimuli to a back‐projection screen mounted inside the MR tube and visible through a mirror mounted inside the head coil. Presentation timing was controlled and triggered by the acquisition of fMRI images. For the campus experiment and the face/scene localizer scans, 238 and 242 volumes were acquired, respectively. Structural images were collected for each subject using a sagittal magnetization‐prepared rapid acquisition gradient echo (MPRAGE) T1‐weighted sequence with the following imaging parameters: 176 slices, in‐plane resolution = 0.5 × 0.5 mm, slice thickness = 1 mm, TR = 2 s, TE = 4.38 ms, and flip angle = 8°.

### 2.4 Image analysis

Image preprocessing was performed using the SPM12 (version: 7771) software package (Wellcome centre for Human Neuroimaging) and individual cortical surfaces were reconstructed using FreeSurfer 5.1 (http://surfer.nmr.mgh.harvard.edu/). Structural images were analyzed following the “recon‐all” fully automated processing pipeline implemented in FreeSurfer 5.1 (Dale et al., 1999; Desikan et al., 2006; Fischl, Sereno, & Dale, 1999; Fischl, Sereno, Tootell, & Dale, 1999) to obtain a surface representation of each individual cortical hemisphere in a standard space. The surface reconstructions were transformed to the symmetrical FS‐LR space (Van Essen et al., 2012) using tools in the Connectome Workbench software (https://www.humanconnectome.org/software/get-connectome-workbench), resulting in surface meshes with approximately 74k nodes per hemisphere.

In each scan, we discarded the first four volumes to exclude non‐steady‐state scans. Images were realigned to the first functional volume of each session and were coregistered to the skull‐stripped anatomical image using SPM12. Functional data were then resampled to the individual cortical surface using ribbon‐constrained resampling as implemented in Connectome Workbench (Glasser et al., 2013), and finally smoothed along the surface with an iterative procedure emulating a Gaussian kernel with a 6 mm full width at half‐maximum (FWHM). Then, we analyzed functional images for each participant separately on a vertex‐by‐vertex basis, according to the general linear model (GLM).

For the face/scene localizer, face and scene blocks were modeled as box‐car functions, convolved with a canonical hemodynamic response function. Fixation periods were not explicitly modeled as GLM regressors and were treated as part of residual variance. In the campus experiment, each trial was modeled as a canonical hemodynamic response function time‐locked to the trial onset and by modeling each trial as a function of the task (perception and imagery). Question trials were modeled separately and not included in further analyses. As a nuisance regressor, we included the framewise displacement (FD), a subject‐specific time‐series index of the overall estimate of movement over time (Power et al., 2012). We computed FD as the sum of the absolute temporal derivatives of the six head‐movement‐related parameters, three for translations and three for rotations.

### 2.5 ROI definition and time‐series extraction

In the first data set, 44 out of the 77 subjects underwent both resting‐state scans and localizer scans. Using the localizer scan, we defined four scene‐selective regions on the cortical surface of each individual hemisphere: the OPA, the anterior parahippocampal place area (aPPA), the posterior parahippocampal place area (pPPA), and the RSC, which were strongly and bilaterally activated as shown by the scene versus face T‐contrast. We used a threshold‐free T‐map after the removal of the deactivations (i.e., *T* values < 0) of the contrast we were interested in (scenes > faces) to select single activation peaks of ROIs and their neighborhood (for a maximum of 400 cortical vertices) using a watershed segmentation algorithm as applied to surface meshes (Mangan & Whitaker, 1999). From the individual data‐driven regions detected by the watershed transform, we selected as individual ROIs the ones which mostly met the following anatomical landmarks: the OPA was mapped close to the TOS, RSC was mapped in the retrosplenial/parieto‐occipital sulcus at the junction with the anterior calcarine sulcus. Regarding PPA, two distinct foci of activation—the aPPA and the pPPA—were mapped along the posterior–anterior/medio‐lateral axis of the posterior parahippocampal cortex, in line with previous reports (Baldassano et al., 2013; Baldassano, Esteva, et al., 2016). Then, we averaged the 44 individual ROIs to create six probabilistic ROIs (OPA, aPPA, pPPA, RSC, aHC, and pHC) used for the rs‐fc analysis of 77 subjects.

Moreover, we defined a hippocampal ROI including all CA fields and the subiculum but not the entorhinal cortex from the automated anatomical reconstruction provided by FreeSurfer, and we divided the HC into an anterior (aHC) and a posterior (pHC) ROI by splitting at *z* = −9 (Morgan et al., 2011). Finally, the primary visual area (V1) was defined as a single surface‐ROI taken from a parcellation atlas (Van Essen et al., 2012) for all subjects. Following the same procedures, in the second data set, we defined the four scene‐selective regions (OPA, aPPA, pPPA, and RSC) on the individual cortical surfaces of the 14 subjects using the localizer images, then we reconstructed the anterior and the posterior hippocampus of each subject and we used the primary visual area V1 as defined from the atlas.

### 2.6 rs‐fc analysis

To account for spurious fluctuations, the resting‐state data were first modeled with a GLM containing six head motion regressors (three translational and three rotational), cerebrospinal fluid signal, and white matter signal. Before entering the GLM, data were band‐pass filtered with a low‐pass cutoff frequency of 0.1 Hz to include only slow BOLD fluctuations (Fox & Raichle, 2007) and a high‐pass cutoff frequency of 0.01 Hz. The time series from the seven ROIs (i.e., OPA, aPPA, pPPA, RSC, aHC, pHC, and V1) were then extracted as the across‐vertices averages of the residual time courses from the GLM, that is, after removing the effect of the sources of spurious variance modeled in the GLM, and a functional connectivity analysis using a seed‐to‐seed approach was performed.

For each subject and each pair of ROIs, the partial Pearson correlation coefficient between the two corresponding regional BOLD time courses was calculated. After transforming correlation coefficients to *z* values using the Fisher transform, one‐sample, one‐tailed *t*‐tests were computed on *z* values, separately for each pair of regions, to assess whether correlation coefficients were significantly higher than 0. A Bonferroni correction for multiple comparisons was applied (*p* < .01 Bonferroni corrected for the 21 connections included in the analysis).

### 2.7 DCM analysis

To infer whether perceiving and imagining a familiar scene differently affected the mutual influences of one region on another among the selected brain areas, we used DCM (Friston et al., 2003). DCM is a framework for making inferences about the directed causal influences of one region on another, or in other words to study the effective connectivity among brain regions. According to DCM, the changes in neural activity ($\dot{z}$) during the experiment can be modeled using the following equation:

$$\dot{z}=\left(A+\sum_{k=1}^{n}u_{k}B^{k}\right)z+\mathrm{Cu},$$

where parameters in matrix A specify the intrinsic connectivity between nodes (i.e., regions) at the baseline; the parameters in matrices B represent the modulation of effective connectivity of one node on another due to experimental input (*k* = 1…n); *u* stands for each experimental input and C specifies a matrix where each parameter shows the sensitivity of a region to the driving input stimulus (Zeidman, Jafarian, Corbin, et al., 2019).

The strength of neural connectivity is represented by DCM parameters that have a neurobiological interpretation: positive values of A‐matrix parameters are interpreted as excitatory influences of one area on another, while negative values are interpreted as inhibitory influences. Similarly, B‐matrix parameters reflect the increase (positive value) or decrease (negative value) of the coupling from one region to another. In both cases, the parameters represent the rate of change of one region's activity caused by activity in other regions. The absolute parameter value defines the strength of the neural connection. The DCM framework includes a neural model and a hemodynamic model—which specifies how hidden neural activity is mapped to BOLD responses.

### 2.8 DCM: Model specification

As part of the DCM analysis, a model architecture needs to be built. This entails specifying which parameters should be switched on (i.e., informed by the data) and which ones should be switched off (i.e., with a prior expectation of zero) (Zeidman, Jafarian, Corbin, et al., 2019). We defined A‐matrix constraints based on anatomical studies on homologous primate brain regions.

Regarding the C‐matrix, which model the sensitivity of a region to the driving input stimulus, perception trials started with a visual cue, so we set V1 as the region driving the input. We assumed that imagery could be driven internally but we had no a priori hypothesis regarding which region drives imagery. Thus, we chose to include in the C matrix all the possible nodes included in our DCM analysis except for V1 and OPA since our group GLM analysis might have not been sensitive enough to capture activation in these regions. We then selected the model with the highest free energy across participants (using a fixed effects approach) to define which region was the most sensitive to the driving input. In other words, the best model was considered to be the one with the highest (i.e., more positive) free energy, that is, the model that offers the most accurate and least complex explanation of the data (Zeidman, Jafarian, Corbin, et al., 2019). The perception input stimuli were always set on V1 and imagery input stimuli were set on each region one at time. The imagery input stimuli on anterior hippocampus were considered together with the posterior hippocampus.

In the B‐matrix, representing the condition‐dependent modulations of connections, we allowed all connections to be modulated by the perception condition, whereas we did not allow connectivity with V1 and OPA to be modulated by the imagery condition since these regions were not activated in the group GLM analysis. For each subject and region, we extracted a single representative time series, retaining the first principal component of adjusted data. Then, we specified and inverted a DCM with all possible connections among regions, that is, the full model, for each subject. We checked that each subject‐specific DCM met the following criteria: (1) the variance explained by the model was at least 10%, as an index of the success of model inversion (Zeidman, Jafarian, Corbin, et al., 2019); (2) at least one connection had a connection strength greater than 1/8 Hz; (3) at least one parameter was effectively estimated (based on Kullback–Leibler divergence of posterior from prior distribution). One subject did not meet these criteria and, thus, was excluded from further analysis. Then, we performed a second‐level analysis (between subjects) on 13 participants over the first‐level DCM parameter estimates.

Finally, we used parametric empirical Bayes (PEB; Friston et al., 2016) to estimate parameters at the group level. Only parameters with strong evidence (i.e., posterior probability higher than 95%) were considered significant. Briefly, the PEB analysis entails the assessment of the commonalities (and differences) among subjects in the effective connectivity domain. PEB is a Bayesian hierarchical model used for group‐level inference. Also, a Bayesian model reduction (BMR) and Bayesian model average (BMA; Friston et al., 2016) were performed after running the full PEB model. By combining BMR with a greedy search, parameters that did not increase the model evidence were efficiently pruned out (Friston et al., 2016; Friston & Penny, 2011). A BMA was performed to average the parameters across models, weighted by the evidence of each model (Friston et al., 2016; Penny et al., 2006), and only parameters whose posterior probability was higher than 95% were selected.

We carried out two separate PEB analyses, one for the A matrix and one for B and C matrices to avoid dilution of evidence effect, and to reduce the search space (Zeidman, Jafarian, Seghier, et al., 2019). Furthermore, since we were only interested in the group means, we did not model other between‐subject effects. Finally, we computed the probability of a difference over parameters of shared connections (with a posterior probability higher than 95%), thus excluding the ones between V1 and OPA, between perception and imagery by using Bayesian contrasts as implemented in SPM12 (https://github.com/spm/spm/blob/main/spm_dcm_peb_con.m).

## 3 RESULTS

### 3.1 ROIs selection and whole‐brain analysis

**FIGURE 1:**
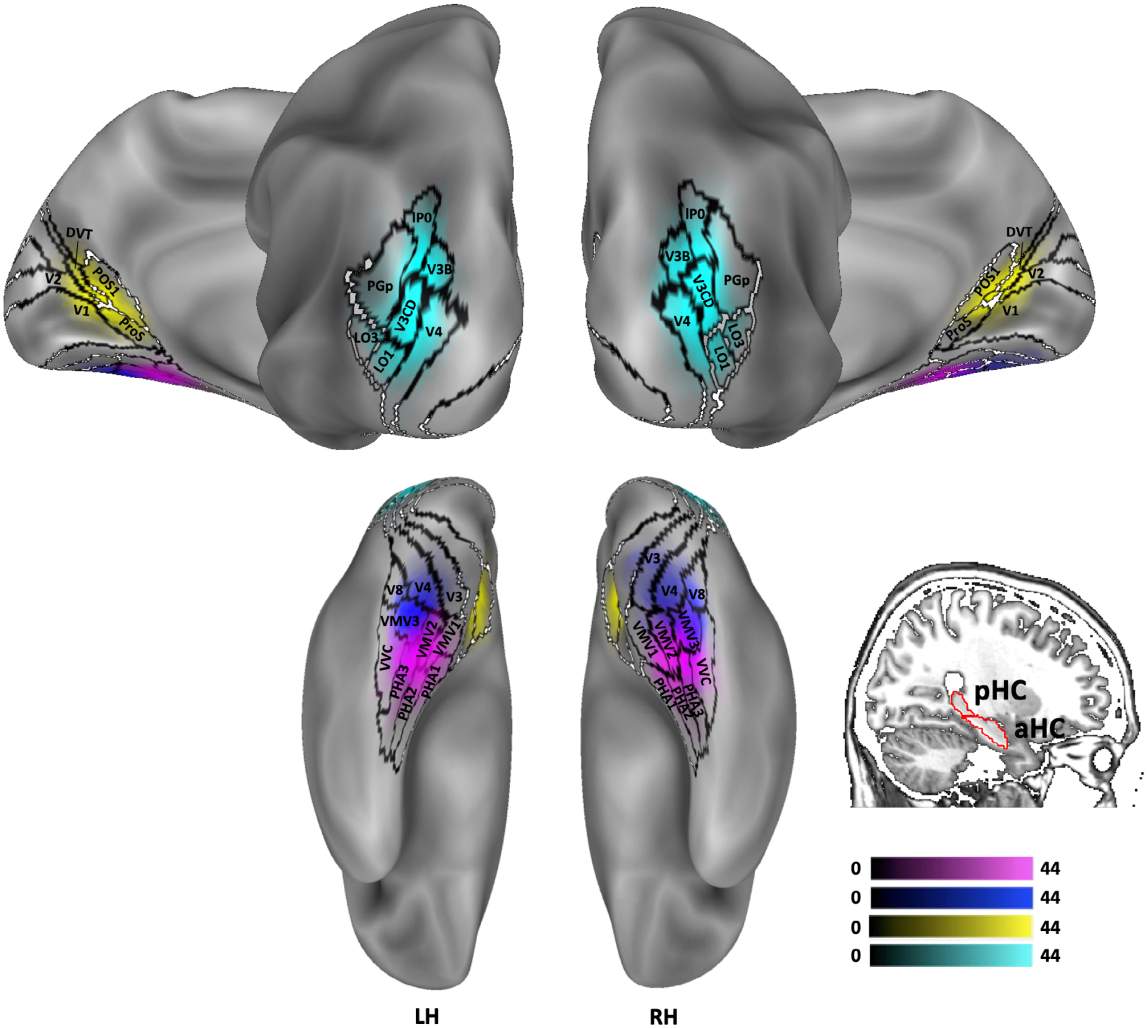
Anatomical location of the regions of interest (ROIs). Probabilistic ROIs of the 44 subjects are overlaid with regions of Human Connectome Project Multi‐modal Parcellation (HCP‐MMP) atlas (Glasser et al., 2016) and shown in posterior and medial (on the top) and inferior (on the bottom) view. The anterior parahippocampal place area (aPPA) is shown in magenta, the posterior parahippocampal place area (pPPA) is shown in blue, the retrosplenial complex (RSC) in yellow, the occipital place area (OPA) in light blue. The color saturation represents the proportion of subjects whose region included that node: the higher the color saturation, the higher the probability that the node is commonly activated across the 44 individual ROIs. Borders of HCP‐MMP regions are shown in black, whereas the borders of the anterior (aHC) and posterior hippocampus (pHC) are marked in red.

**FIGURE 2:**
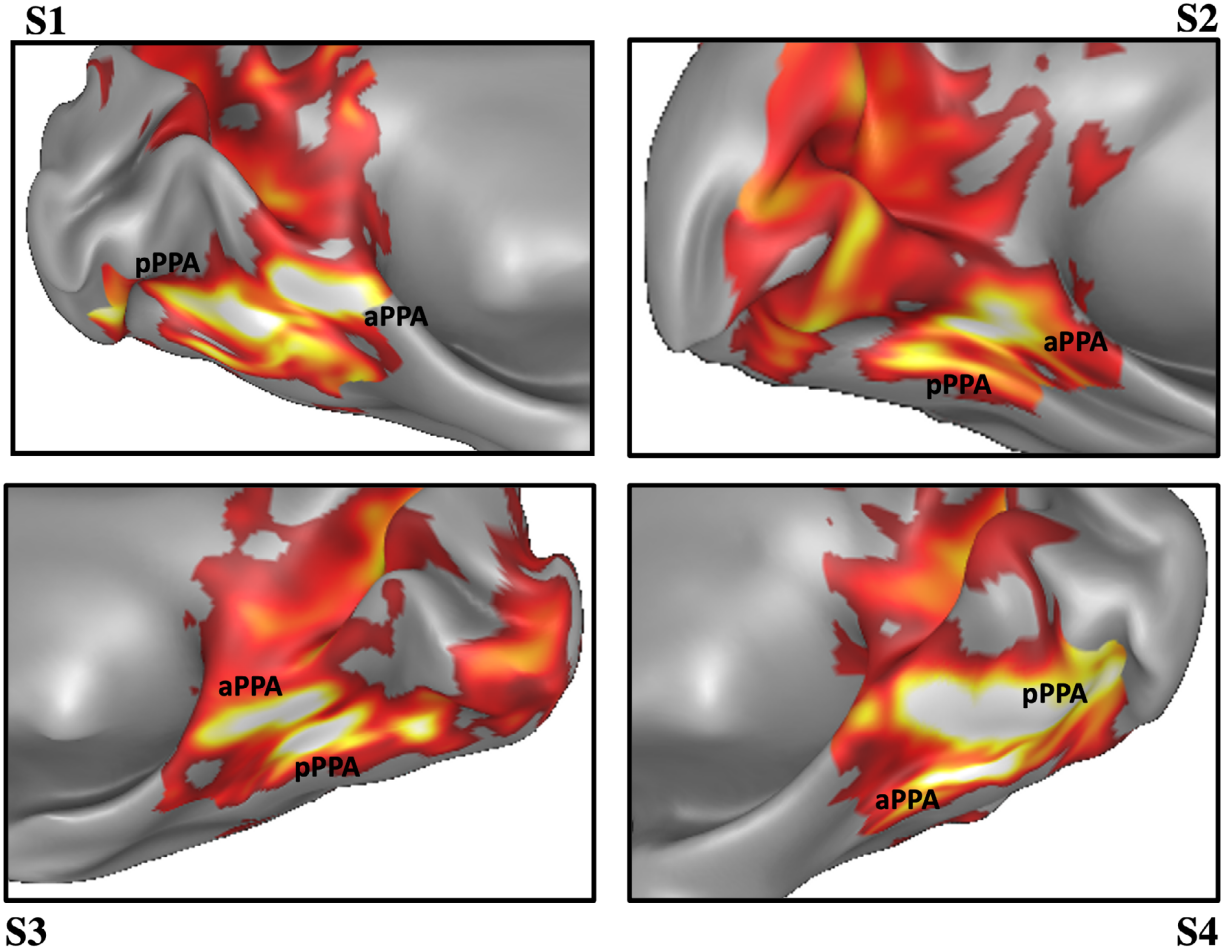
Representative parahippocampal place area (PPA) regions of interest (ROIs). Representative PPA, anterior (aPPA), and posterior (pPPA) of four subjects (S1–S4) in the left hemisphere (upper panel) and right hemisphere (lower panel).

From the localizer of the first data set, we defined four scene‐selective regions (OPA, aPPA, pPPA, and RSC) determined as the regions responding more to scene than to face stimuli. Regions were defined on the cortical surface of 44 subjects and then four probabilistic ROIs were calculated by averaging individual ROIs across subjects. All the probabilistic regions are available online (https://github.com/maggiu/TopoimageDCM_data.git). Figure 1 shows the resulting probabilistic ROIs overlaid with regions of Human Connectome Project Multi‐modal Parcellation (HCP‐MMP) atlas (Glasser et al., 2016), together with the V1, pHC, and aHC ROIs. The OPA plenty overlapped with V3CD, V3B, and IP0, as previously observed (Sulpizio et al., 2020), and partially overlapped with PGp, LO1, LO3, and V4, whereas the RSC overlapped with the prostriate region ProS and DVT as previously found (Rolls, Deco, et al., 2023; Rolls, Wirth, et al., 2023; Sulpizio et al., 2020). Importantly, consistent segregation into two PPA activation foci, an anterior and a posterior, was observed. The posterior PPA was mainly centered on MVM3 and on the most anterior part of V4 and V8; the anterior PPA included portions of parahippocampal areas PHA1, PHA2, PHA3, VMV2, and VVC (Glasser et al., 2016). Figure 2 shows the anterior PPA and the posterior PPA of four representative subjects.

**FIGURE 3:**
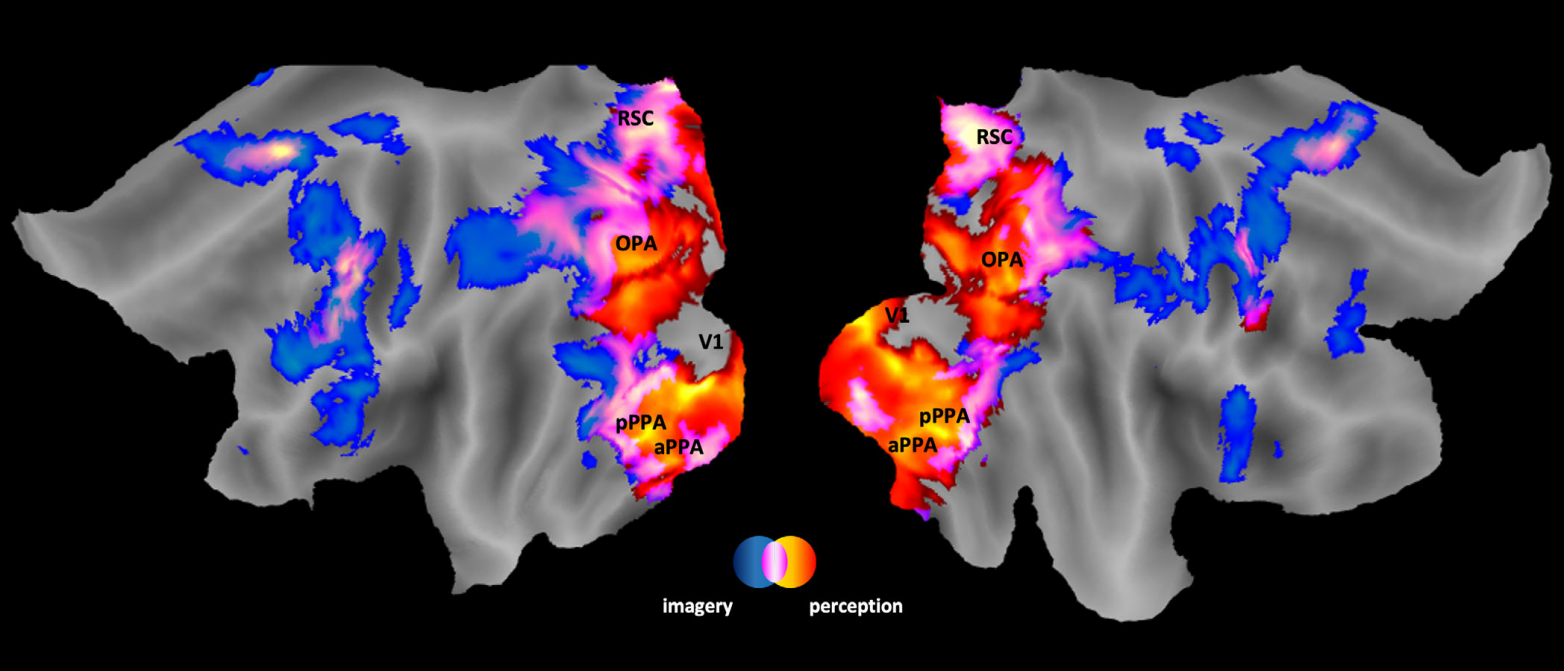
Whole‐brain results of the campus experiment. Superimposition of the group activation map respectively of the perception > fix (from red to yellow) and of the imagery (from blue to turquoise) t‐contrast; commonly activated areas are displayed in color saturation from pink to white representing the degree of overlap between perception and imagery condition, the higher the overlap the higher the saturation (white). The maps are overlaid into the flattened Conte69 atlas (Van Essen et al., 2012) of the left and right hemispheres. The main activation is labeled as follows: V1, primary visual area; OPA, occipital place area, pPPA, posterior parahippocampal place area; aPPA, anterior parahippocampal place area; RSC, retrosplenial complex.

A detailed description of the whole‐brain effects of the two experimental conditions in the campus experiment (perception and imagery condition) is provided by Boccia et al. (2017). In Figure 3, the overlap of the group activation maps of the perception and imagery condition is shown. The activation map was overlaid onto the flattened Conte69 atlas. Specifically, the perception of familiar scenes (perception > fix t‐contrast) revealed a wide set of activations spanning from the occipital to the frontal lobe and encompassing bilaterally the calcarine cortex, the RSC, the fusiform gyrus, the middle frontal gyrus, the middle occipital gyrus, and the precentral gyrus. In contrast, the imagery of familiar scene location (imagery > fix t‐contrast) activated the RSC, the supplementary motor area, the precentral gyrus, the superior parietal lobule, and the inferior temporal gyrus. In both perception and imagery conditions, the RSC, the inferior temporal gyrus, the supplementary motor area, the precentral gyrus, and the superior parietal lobule were active. In summary, we detected in both tasks an activation map that included each of our ROIs (i.e., the bilateral RSC, pPPA and aPPA, aHC, and pHC). An accurate description of region coordinates activated during imagery, perception, and both conditions are given by Boccia et al. (2017).

### 3.2 Resting‐state functional connectivity

The analysis of the correlation patterns of spontaneous fluctuations during resting state was used to assess the connectivity preference for the scene‐selective regions (OPA, aPPA, pPPA, and RSC), the primary visual area (V1), and the HC to reveal the existence of functional networks linking our ROIs (van den Heuvel & Hulshoff Pol, 2010).

**FIGURE 4:**
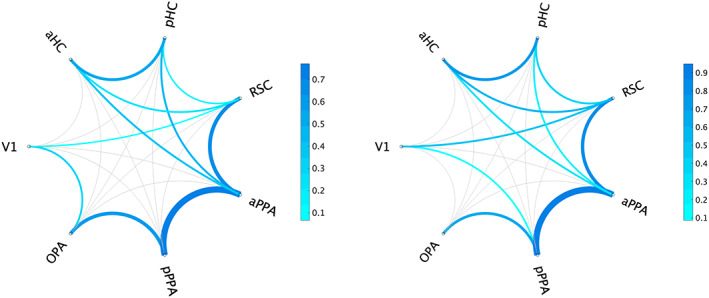
Graphical representation of the resting‐state functional connectivity across regions of interest (ROIs). Significant correlations (*p* < .01, corrected) are shown in shades of blue proportionally to the strength of each connection as represented by the average, normalized correlation coefficients ranging from 0.1 to 0.7 in the left hemisphere and from 0.1 to 0.9 in the right hemisphere. Non‐significant correlations (*p* > .00047) are shown in light gray.

Results of the rs‐fc analysis among V1, OPA, RSC, aPPA, pPPA, aHC, and pHC on 77 subjects revealed the existence, in both hemispheres, of significant (*p* < .01, Bonferroni corrected, i.e., *p* < .00047) functional connections between V1 and RSC, OPA and pPPA, RSC and both aHC and pHC, RSC and aPPA, aPPA and pPPA, aPPA and both aHC and pHC, aHC and pHC. In the left, but not in the right hemisphere, a significant coupling was found between V1 and OPA. Conversely, in the right but not in the left hemisphere, a significant coupling was found between V1 and pPPA. For conciseness, *Z*‐transformed connectivity values along with *t* values and uncorrected *p* values of the one‐sided one‐sample *t* test are reported in Table 1. Mean *z* values of significant connections (*p* < .00047) are shown in bold. Also, results are schematically reported in Figure 4.

**TABLE 1:** Results of the resting‐state functional connectivity analysis.

	V1	OPA	pPPA	aPPA	RSC	pHC	aHC
V1		*Z* = 0.06	***Z* = 0.08**	*Z* = −0.13	***Z* = 0.12**	*Z* = 0.03	*Z* = 0.02
		*t* = 1.77	*t* = 3.60	*t* = −4.61	*t* = 4.24	*t* = 2.17	*t* = 1.25
		*p* = 3.98 × 10^−2^	*p* = 2.74 × 10^−4^	*p* = 1.00 × 10^0^	*p* = 3.03 × 10^−5^	*p* = 1.66 × 10^−2^	*p* = 1.06 × 10^−1^
OPA	***Z* = 0.13**		***Z* = 0.28**	*Z* = 0.04	*Z* = −0.004	*Z* = −0.02	*Z* = −0.07
	*t* = 4.00		*t* = 11.92	*t* = 1.92	*t* = −0.22	*t* = −1.23	*t* = −4.18
	*p* = 7.24 × 10^−5^		*p* = 2.20 × 10^−19^	*p* = 2.92 × 10^−2^	*p* = 5.87 × 10^−1^	*p* = 8.90 × 10^−1^	*p* = 1.00 × 10^0^
pPPA	*Z* = 0.02	***Z* = 0.37**		***Z* = 0.94**	*Z* = −0.23	*Z* = −0.07	*Z* = −0.02
	*t* = 1.10	*t* = 12.86		*t* = 39.41	*t* = −8.78	*t* = −4.35	*t* = −1.45
	*p* = 1.37 × 10^−1^	*p* = 4.50 × 10^−21^		*p* = 1.20 × 10^−52^	*p* = 1.00 × 10^0^	*p* = 1.00 × 10^0^	*p* = 9.25 × 10^−1^
aPPA	*Z* = −0.03	*Z* = −0.01	***Z* = 0.77**		***Z* = 0.40**	***Z* = 0.09**	***Z* = 0.11**
	*t* = −1.75	*t* = −0.59	*t* = 24.79		*t* = 13.49	*t* = 3.98	*t* = 6.50
	*p* = 9.58 × 10^−1^	*p* = 7.24 × 10^−1^	*p* = 1.83 × 10^−38^		*p* = 3.62 × 10^−22^	*p* = 7.57 × 10^−5^	*p* = 3.78 × 10^−9^
RSC	***Z* = 0.06**	*Z* = 0.07	*Z* = −0.14	***Z* = 0.40**		***Z* = 0.12**	***Z* = 0.14**
	*t* = 3.57	*t* = 2.32	*t* = −6.11	*t* = 15.31		*t* = 5.66	*t* = 7.31
	*p* = 3.08 × 10^−4^	*p* = 1.13 × 10^−2^	*p* = 1.00 × 10^0^	*p* = 3.04 × 10^−25^		*p* = 1.23 × 10^−7^	*p* = 1.12 × 10^−10^
pHC	*Z* = 0.005	*Z* = −0.04	*Z* = −0.04	***Z* = 0.14**	***Z* = 0.07**		***Z* = 0.29**
	*t* = 0.31	*t* = −2.82	*t* = −2.35	*t* = 5.51	*t* = 4.01		*t* = 12.62
	*p* = 3.77 × 10^−1^	*p* = 9.97 × 10^−1^	*p* = 9.89 × 10^−1^	*p* = 2.33 × 10^−7^	*p* = 6.83 × 10^−5^		*p* = 1.21 × 10^−20^
aHC	*Z* = 0.03	*Z* = −0.06	*Z* = −0.009	***Z* = 0.14**	***Z* = 0.12**	***Z* = 0.28**	
	*t* = 1.67	*t* = −3.28	*t* = −0.52	*t* = 7.77	*t* = 5.93	*t* = 13.61	
	*p* = 4.90 × 10^−2^	*p* = 9.99 × 10^−1^	*p* = 6.99 × 10^−1^	*p* = 1.47 × 10^−11^	*p* = 4.12 × 10^−8^	*p* = 2.21 × 10^−22^	

*Note*: Connections between regions in the right hemisphere are shown above the main diagonal, whereas the connections between regions in the left hemisphere are shown below the main diagonal. For each connection between each pair of regions, *Z*‐transformed connectivity values, *t* values and uncorrected *p* values of the one‐sided one‐sample *t* test are shown. Mean *z* values of significant connections (*p* < .00047) are bolded.

In line with previous studies (Baldassano, Esteva, et al., 2016; Baldassano, Fei‐Fei, & Beck, 2016), the two PPA portions, that is, the anterior and the posterior PPA, showed a preferential co‐activation with medially located regions such as RSC, and the HC and with dorso‐lateral regions such as OPA, respectively. Similar connectivity patterns were observed in anatomical studies on primates defining two preferential pathways for TF and TH/TFO (Kravitz et al., 2011).

### 3.3 DCM model specification

**FIGURE 5:**
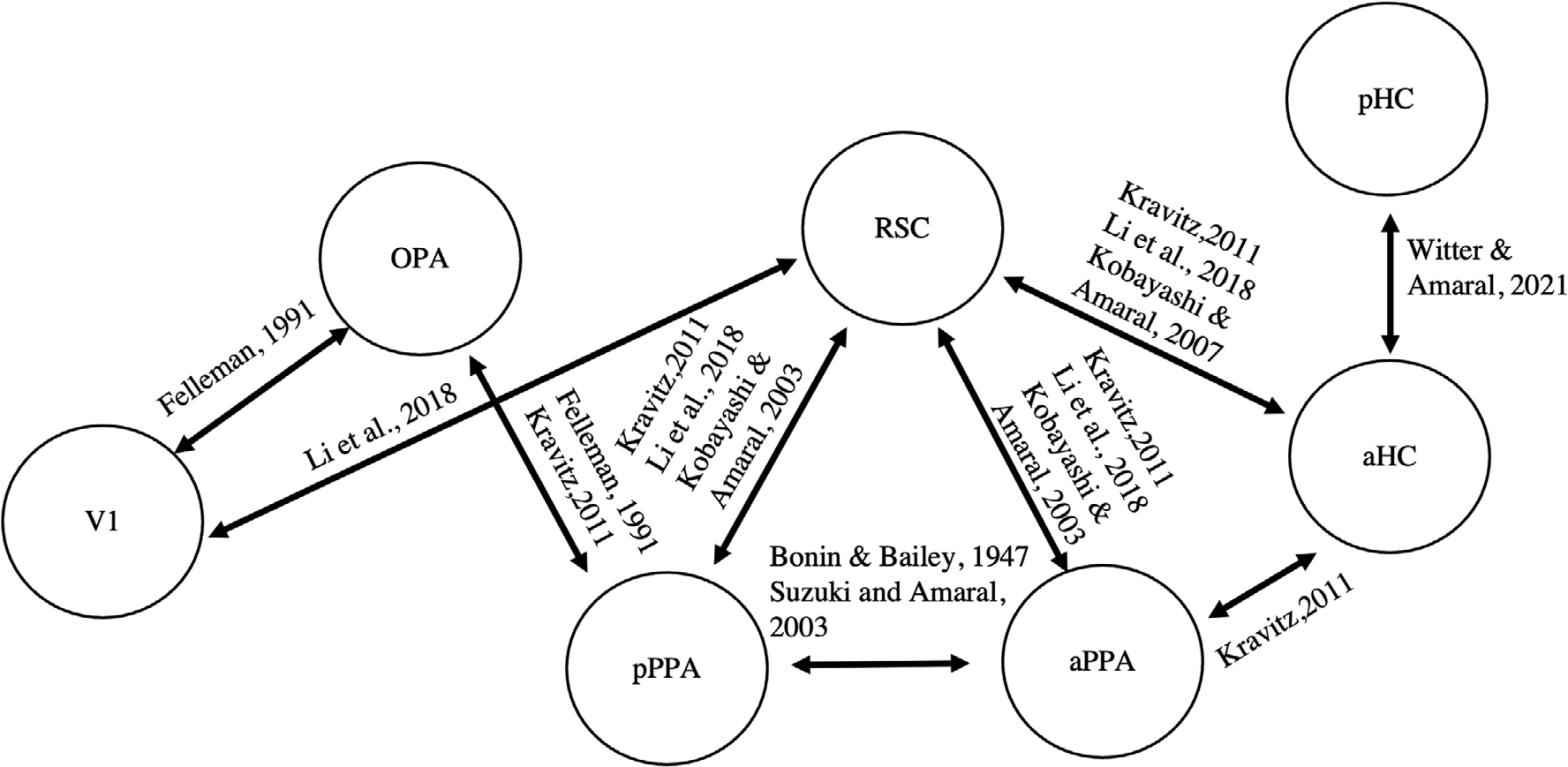
Dynamic causal modeling (DCM) model scheme. Black solid arrows show the constraints of the DCM model among regions of interest based on tracer studies in macaques whose references are written above the arrows. The regions included in the model were the primary visual area (V1), the occipital place area (OPA), the posterior parahippocampal place area (pPPA), the anterior parahippocampal place area (aPPA), the retrosplenial complex (RSC), the anterior hippocampus (aHC), and the posterior hippocampus (pHC).

To study the effective connectivity among scene‐selective regions, V1, and HC during the perception and the imagery of familiar scenes, we based our model on previous results of anatomical studies on macaques. Indeed, the regions we selected in this study entail scene‐perception and navigational tasks and are the human homolog of macaque brain regions involved in the spatial‐navigation pathway. Therefore, we identified the endogenous connections of A‐matrix using results from anatomical studies in macaques reported in Figure 5, allowing the bi‐directional connections between V1 and OPA (Felleman & Van Essen, 1991), V1 and RSC (Li et al., 2018), OPA and pPPA (Kobayashi & Amaral, 2003), RSC and pPPA (Kobayashi & Amaral, 2003; Kravitz et al., 2011; Li et al., 2018), RSC and aPPA (Kobayashi & Amaral, 2003; Kravitz et al., 2011; Li et al., 2018), aPPA and pPPA (Suzuki & Amaral, 2003; von Bonin & Bailey, 1947), RSC and aHC (Kobayashi & Amaral, 2007; Kravitz et al., 2011; Li et al., 2018), aHC and pHC (Witter & Amaral, 2021).

### 3.4 Parametric empirical Bayes

**FIGURE 6:**
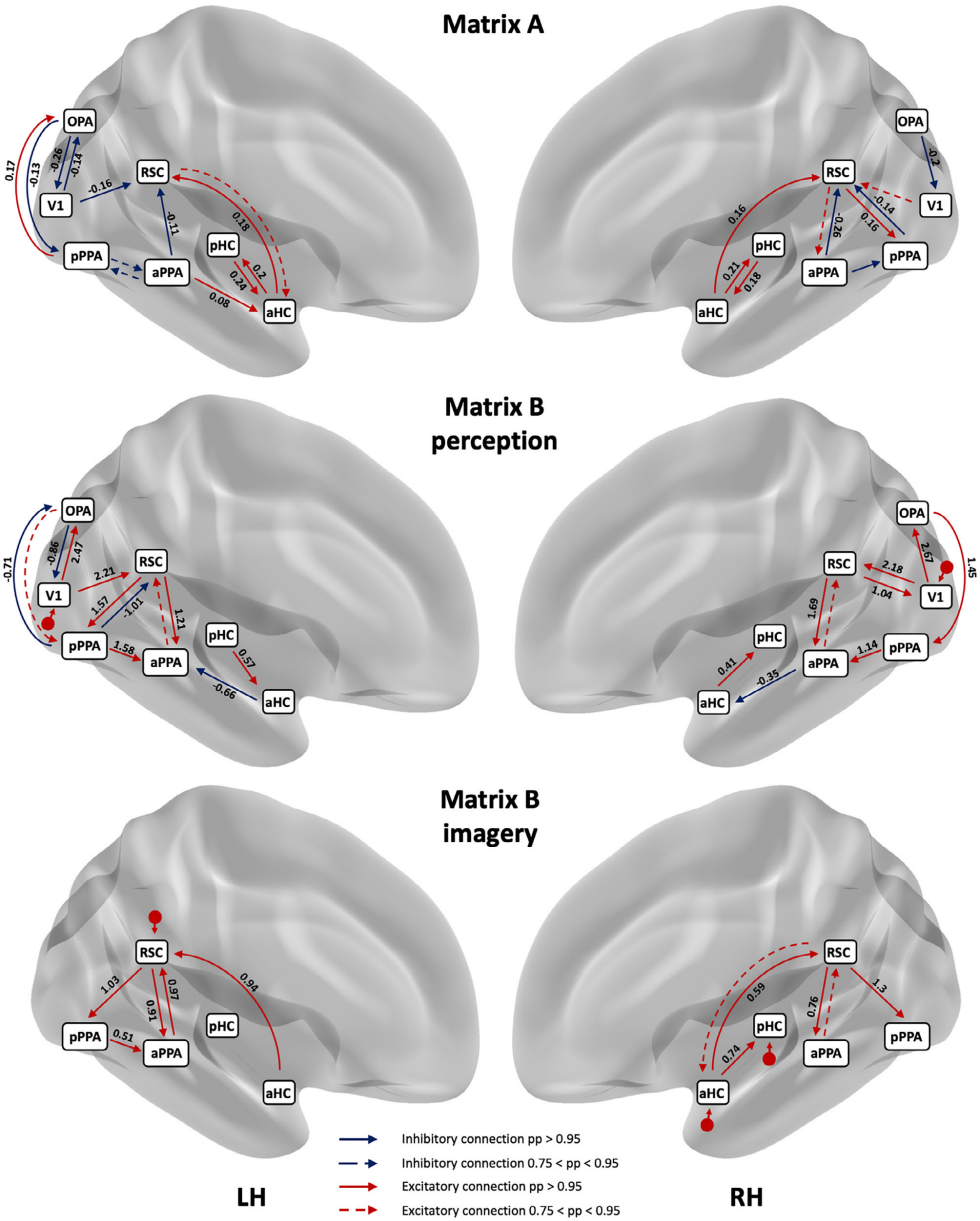
Schematic representations of A‐matrix (upper panel) and B‐matrices (middle and lower panel) in the left and the right hemisphere: red solid arrows represent excitatory connections and blue solid arrows represent inhibitory connections. Connection parameters with a posterior probability below the 0.95 threshold but ranging from 0.75 to 0.95 are reported with dotted arrows. Values of connection strengths exceeding a posterior probability of 0.95 are also provided above the arrows. The input stimulus is represented by a small red arrow. In the left hemisphere (LH) and the right hemisphere (RH), during perception the input stimulus is set on V1, whereas during imagery, the input stimulus is set on RSC in the left hemisphere and on aHC and pHC in the right hemisphere. Regions are labeled as follows: primary visual area (V1), Occipital Place Area (OPA), anterior Parahippocampal Place Area (aPPA), posterior Parahippocampal Place Area (pPPA), Retrosplenial Complex (RSC), anterior hippocampus (aHC), and posterior hippocampus (pHC).

PEB analysis revealed that the intrinsic connectivity (A‐matrix) of our model was similar in both the right and left hemispheres (see the upper panel of Figure 6). Indeed, at baseline, antero‐posterior directed inhibitory influence was present: the OPA had an inhibitory effect on the primary visual area V1. Similarly, the anterior PPA had an inhibitory effect on both the posterior PPA and RSC. In contrast, the HC—the anterior and the posterior—showed a positive effect on the regions with which it is connected. The posterior hippocampus and the anterior hippocampus had a bidirectional influence, but only the anterior hippocampus excited the RSC. However, some differences were present between the left and the right hemisphere: left V1 inhibited both left OPA and left RSC, whereas right V1 had no suprathreshold connection with right OPA but excited the right RSC, though the posterior probability was below the threshold (pp = .83). In the left hemisphere, OPA inhibited pPPA and pPPA excited OPA, whereas in the right hemisphere no suprathreshold values were obtained from OPA to pPPA and the pPPA inhibited OPA. Moreover, in the left hemisphere, aPPA excited aHC, whereas this connection was missing in the right hemisphere. In the right hemisphere, the RSC excited pPPA and pPPA inhibited RSC, while these connections were missing in the left hemisphere (see the upper panel in Figure 6).

The most interesting results concerned the input stimulus (C‐matrix) and modulatory parameters (B‐matrix) in both perception and imagery conditions. We took into account that, according to the macaque model, brain connectivity during perception would involve the circuit starting from the primary visual area V1 and proceed forward towards temporo‐medial regions. As opposed to perception, during imagery, we set the input stimulus on each region of the model except for V1 and OPA since these regions were not active in the voxel‐wise map (imagery > fix t‐contrast). Then, we chose the model offering the best trade‐off between accuracy and complexity, which was the one with the highest free energy cross participants. The model which best explained the data was the one with the perception input stimulus on V1 and imagery input stimulus on RSC in the left hemisphere (*F* = −140,782,4) and the hippocampus in the right hemisphere (*F* = −133,512,8) as shown in Table 2.

**TABLE 2:** Free energy of DCM models.

Input stimulus		Free energy	
Perception	Imagery	Left hemisphere	Right hemisphere
V1	HC	−141,076,3576	**−133,512,8967**
V1	RSC	**−140,782,4527**	−145,703,4048
V1	PPA	−141,118,3437	−145,755,2487

*Note*: The regions shown on the left side of the table are those on whom the input stimulus of perception and imagery was modeled. The primary visual area (V1) was chosen as this region was more active compared to the others during the perception condition, while the hippocampus (HC; i.e., both the anterior and the posterior hippocampus), the retrosplenial complex (RSC) and the parahippocampal place area (PPA) were selected as candidate input regions for imagery condition. The summed free energy across participants is shown on the right. The highest free energy is highlighted in bold and corresponds to the model in which the perception input stimulus was set to V1 and the imagery input stimulus was set to RSC and HC in the left and the right hemisphere, respectively.

We were mainly interested in modulations of forward and backward connections captured by the B‐matrices. As previously said, we modeled all connections of the B‐matrix in the perception condition, whereas we excluded the connectivity with V1 and OPA in the imagery condition. We will discuss results in the left hemisphere first. Note that, excitation indicates that a region increases the signal in another region with respect to the parameter value at rest (A‐matrix), while inhibition indicates the opposite. For a schematic representation of effective connectivity during perception and imagery, see the middle and lower panels of Figure 6. In the left hemisphere, during perception, V1 increased the signal in OPA with a connection strength of 2.36 Hz, which in return inhibited V1 (−0.83 Hz), and RSC (2.2 Hz). Concurrently, RSC excited the pPPA (1.58 Hz) and the aPPA (1.21 Hz), while OPA excited the pPPA to a lesser extent with a posterior probability (pp) of 77%. The pPPA excited aPPA (1.6 Hz) and inhibited RSC (−0.99 Hz) and OPA (−0.7 Hz). Then, the posterior hippocampus excited the anterior hippocampus (0.58 Hz) and aHC inhibited aPPA (−0.67 Hz). Note that in the left hemisphere, as well as the right one, aPPA excited RSC but its posterior probability did not exceed the threshold (LH: pp = 88%, connection strength = 0.39; RH: pp = 79%, connection strength = 0.3).

During imagery, the model that best explained the observed responses was the one where the input stimulus was set on the RSC which excited both the anterior (0.91 Hz) and the posterior PPA (1.03 Hz). Concurrently, RSC received an excitatory input from both aHC (0.94 Hz) and aPPA (0.97 Hz). Finally, pPPA excited aPPA (0.51 Hz). Here, the anterior portion of the hippocampus excited RSC (0.93 Hz) and from the RSC the signal excited both aPPA (0.9 Hz) and pPPA (1.02 Hz). Concurrently, the pPPA excites aPPA (0.51 Hz). In the right hemisphere, during perception, V1 excited OPA (2.67 Hz) and RSC (2.18 Hz), while RSC excited V1 (1.04 Hz) and aPPA (1.69 Hz). The OPA excited pPPA (1.45 Hz), pPPA excited aPPA (1.14 Hz), and aPPA inhibited aHC (−0.35 Hz). Finally, aHC excited pHC (0.41 Hz). To a lesser extent, aPPA excited RSC (connection strength = 0.30, pp = 79%). During imagery, a crucial role concerned the connectivity between aHC and RSC. In the right hemisphere, as resulting from the winning model, the input stimulus was set on the hippocampus. The anterior hippocampus excited both pHC (0.74 Hz) and RSC (0.59 Hz), while RSC excited both aPPA (0.76 Hz) and pPPA (1.30 Hz). To a lesser extent, RSC excited aHC (connection strength = 0.33, pp = 89%) and aPPA excited RSC (connection strength = 0.39, pp = 85%).

The probability of a difference, higher than 95%, over parameters during perception and imagery is reported in Table 3. The connection strength during imagery from aHC to RSC was higher in both hemispheres (LH: Ep = −0.94, pp = .99; RH: Ep = −0.91, pp = .99) and the connection from aPPA to RSC was higher in the left hemisphere (Ep = −0.58, pp = .98). On the other hand, the connection strength from pPPA to aPPA is higher during perception in both hemispheres (LH: Ep =1.07, pp = .99; RH: Ep = 1.13, pp = 1.00).

**TABLE 3:** Bayesian contrasts: Statistical tests on Dynamic Causal Modeling parameter estimates (Ep) between imagery and perception in the left (LH) and right hemisphere (RH).

Hemisphere	From	To	Pp	Ep
LH	RSC	pPPA	0.98	0.54
	aPPA	RSC	0.98	−0.58
	aHC	RSC	0.99	−0.94
	aHC	aPPA	0.99	−0.65
	pPPA	RSC	0.99	−1.00
	pPPA	aPPA	0.99	1.07
	pHC	aHC	0.99	0.57
RH	RSC	aPPA	0.99	0.92
	RSC	pPPA	1.00	−1.29
	aPPA	aHC	0.99	−0.35
	aHC	RSC	0.99	−0.91
	pPPA	aPPA	1.00	1.13

*Note*: Positive Ep values stand for a more excitatory connectivity during perception than imagery, whereas negative values stand for a more excitatory connectivity during imagery than perception. The posterior probability (Pp) for each connection, higher than 0.95, is also reported. Regions are labeled as follows: anterior Parahippocampal Place Area (aPPA), posterior Parahippocampal Place Area (pPPA), Retrosplenial Complex (RSC), anterior hippocampus (aHC), and posterior hippocampus (pHC).

## 4 DISCUSSION

In the present study, we aimed at defining functional connectivity at rest and the causal influences between regions mediated by the perception and the imagery of familiar scenes. We tested hypotheses about the cortical connections between V1, OPA, aPPA, pPPA, RSC, aHC, and pHC using rs‐fc analysis and tested alternative models during perception and mental imagery using DCM.

### 4.1 Anterior and posterior PPA: Segregated regions, distinguished networks

As a first step, we functionally defined scene‐selective regions in each individual subject. These regions were mapped as preferring places to faces from an independent localizer. Their location was consistent with previous descriptions (Rolls, Deco, et al., 2023; Rolls, Wirth, et al., 2023; Sulpizio et al., 2020) on the HCP‐MMP atlas (Glasser et al., 2016). Regarding PPA, by using the watershed segmentation algorithm as applied to surface meshes (Mangan & Whitaker, 1999), a segregation in two PPA—an anterior and a posterior portion—responding more to scenes than faces was found in all participants. Intriguingly, we studied the functional profile at rest of these regions and found that the anterior PPA and the posterior PPA were differently connected with the rest of the network. Indeed, partial correlations on rs‐fc data revealed that the anterior PPA was connected with both the RSC and the anterior and posterior hippocampus, whereas the posterior PPA was connected with OPA in both the left and the right hemispheres. This is in line with previous studies on humans (Baldassano et al., 2013; Baldassano, Esteva, et al., 2016; Baldassano, Fei‐Fei, & Beck, 2016) and consistent with the macaque literature, which shows the existence of a preferential signal pathway from visual areas to the posterior portion of the temporal area TFO, and interconnections between medial areas and TF/TH (Kravitz et al., 2011; Sewards, 2011).

### 4.2 Effective connectivity in the scene‐selective network

Once the functional architecture of the scene‐selective network was established, we extended previous findings by addressing whether the neural communication between the scene‐selective regions (OPA, pPPA, aPPA, and RSC), the primary visual area, and the hippocampus is differently perturbed by two different conditions: the visual perception of familiar scenes, and their visual imagery.

Notably, DCM results on the hierarchical organization at baseline showed a similar network architecture across subjects in both hemispheres. In both hemispheres, V1 and OPA shared inhibitory connections at rest, whereas during perception V1 strongly excited OPA and RSC. Conversely, the connectivity from OPA to V1 in the left hemisphere during perception was still inhibitory but with a higher negative parameter value than at rest. This result could be interpreted as a balance between the two regions of a monodirectional preference route of the signal towards higher cortical areas. During visual perception of familiar scenes, we found strong evidence in favor of a positive signal from V1 to RSC toward aPPA and pPPA, in a similar way in the left and right hemispheres. While no excitatory connections were shared with the hippocampus during perception, the dynamic couplings from the anterior hippocampus toward the RSC were a neural feature of imagery, as resulted from the Bayesian contrast analysis. An interaction between the right HC and the right PPA was previously observed during the recall of the mental images—but not during the visual perception—of familiar landmarks (Boccia et al., 2017). This observation paved the way to the definition of a more detailed signal route since the anterior hippocampus was found to have an excitatory effect on the RSC which in turn excited aPPA and pPPA.

Moreover, it has been speculated that antero‐posterior pathways from the hippocampus to the neocortex are necessary to recall information about the spatial scenes (Kesner & Rolls, 2015; Treves & Rolls, 1994). It is relevant to remember that the stimuli we used were pictures of buildings placed in the university campus and, thus, very familiar to participants. As such, their identity and their location were likely stored in a strong memory trace. Working in concert with other brain regions to form a cognitive map, the hippocampus, specifically the anterior portion, is recruited during the generation of positional signals (Jahn et al., 2009) and the estimation of direction and distance between landmarks (Taube, 2007) even in the absence of a navigational goal (Morgan et al., 2011). It is reasonable to speculate that, in the absence of visual landmarks, the hippocampus enhances its coupling with the RSC to orient the subject's position in space with the aim of converting the allocentric frame of reference (i.e., world‐based) into egocentric (relative to the body or the head) spatial view coordinates. With that said, a right hemisphere dominance was observed during the imagery condition since the input stimulus was more likely to drive the HC in the right hemisphere. This is in line with neuroimaging and lesion studies employing spatial‐navigation and spatial‐memory paradigms since a unique functional role of the right hippocampus was found in navigation accuracy (Maguire et al., 1998, 2000), the retrieval of spatial location (Boccia et al., 2017; Wolbers et al., 2007) together with impaired spatial memory performance in patients with right hemisphere lesions (Gleissner et al., 1998; van Asselen et al., 2006). These pieces of evidence lead to the belief that the right hemisphere is where spatial processing, specifically when supporting navigation and knowledge of spatial locations, is lateralized.

### 4.3 The RSC as the crucial hub for visual perception and imagery

Importantly, during both perception and imagery, the RSC played a crucial role. The RSC was not functionally connected (left hemisphere) or weakly connected (right hemisphere) with parahippocampal regions at rest, but during perception and imagery RSC strongly excited both aPPA and pPPA in the left hemisphere and solely aPPA in the right hemisphere. During both conditions, RSC excited aPPA in both hemispheres, which is similar to what we observed in the rs‐fc and to the model proposed by (Baldassano, Esteva, et al., 2016).

From the present results, RSC appears to be a central hub during visual perception as well as visual imagery, but with different roles. Indeed, this area was informed by the visual signal encoded in the primary visual cortex during perception, and by the memory trace stored in the hippocampus during imagery. Sequential effective connectivity links at rest were previously observed in the “ventromedial visual stream” (Rolls, Deco, et al., 2023; Rolls, Wirth, et al., 2023), in which strong inputs from early visual areas projected to the posterior part of the parahippocampal gyrus and then to the hippocampal memory system. Besides that, the early visual areas showed an effective connectivity with regions in the PCC that, in turn, were coupled with parahippocampal regions (Rolls, Deco, et al., 2023; Rolls, Wirth, et al., 2023).

Also, it is known that one of the spatial cognitive processes attributed to the RSC is to act as a bridge between regions elaborating sensory information acquired from the environment and regions that store an internal picture of the environment (Burgess et al., 2001). As a midline association region, the function of RSC spans from the elaboration of the more basic characteristic of a scene, such as the perception of landmarks, to higher‐level functions such as navigation‐related signal elaboration, including head direction, positional information processing and self‐orientation within the scene (Mao et al., 2018; Sewards, 2011; Sulpizio et al., 2013). Importantly, this information transfer occurs when subjects update their mental representation of the space when they move in an environment as well as when they recall previously encoded spatial information. As previously observed, low‐level visual information is shared by the parietal areas with the retrosplenial area which elaborates spatial scene view information. Such information is then broadcastened to the ventromedial visual regions and the parahippocampal scene area, which in turn connects with the hippocampal system to build feature‐based scene representations (Rolls, Deco, et al., 2023; Rolls, Wirth, et al., 2023). Furthermore, the primate homolog of RSC is part of the macaque area POda which is connected to V6A, one of the regions afferents to V1. As previously proposed (Sewards, 2011), mental images of scenes could be produced by the activation of mnemonic scene representation in area Poda, converted in area V6A, and visually activated in V1. Also, the prostriate region ProS and the visual areas in the parieto‐occipital sulcus (POS1–2) are strictly close to V1 (Figure 1) and share with visual cortical areas V1–V3, V6, and V6A both anatomical, functional and effective connectivity (Rolls, Deco, et al., 2023; Rolls, Wirth, et al., 2023).

Our results demonstrated that beyond similar activation during perception and imagery, the interaction between brain regions involved in navigation at rest and, more importantly, during active tasks exhibits several differences with brain regions involved in perception. Opposite connectivity between perception and imagery in the areas of the ventral visual stream was found: during perception, we observed a postero‐antero progression of the visual stream starting from occipital areas such as V1 and OPA, to temporal areas as PPA and RSC, and finally to HC. Conversely, during imagery, our results suggest the opposite pattern, that is, a top‐down stream that arises from HC and then reaches RSC and PPA.

In the past, DCM has been used to reveal the neural interactions in perception and imagery (Dijkstra et al., 2017; Mechelli, 2004). Also, our group investigated the impact of imagery abilities on dynamic couplings at rest among brain regions involved in the perception of places (Tullo et al., 2022). Compared to these previous works, we expanded the network of regions of which we assessed the involvement in scene perception and navigation, and we compared the effective connectivity during the perception and the imagery of places. Importantly, the anatomical structure of our networks was motivated by regions selected through an independent localizer and by homologies with the macaque navigational pathway. At the same time, we favored a hypothesis‐driven approach in region‐of‐interest selection to limit the combination of DCM models. We are aware that at least during imagery other regions may be involved and further studies are required to evaluate their contribution, in other aspects of imagery or navigational tasks such as, among the others, attentional control.

### 4.4 The contribution of early and high‐order visual areas to visual imagery

Kosslyn's pioneering studies on visual imagery (Kosslyn et al., 1993, 1995) demonstrated that the primary visual regions were activated during visual imagery and, most importantly, pointed out that these areas could be activated depending on the task performed and the stimuli used. In our experiment, subjects stayed with their eyes opened during both visual imagery and fixation. Specifically, during visual imagery subjects looked at the name of the campus building to be imagined, while during baseline a fixation cross was seen. This choice was used to make the visual and the imagery stimulus as similar as possible. As opposed to perception, during imagery, we had to exclude the modulations of connections with OPA and V1, given that we did not record a consistent activation of these areas during imagery. At the same time, no occipital areas were activated in the group GLM analysis, neither V1 nor OPA. Furthermore, note that we used a task that included recalling the spatial position of the campus building from the perspective of the subject positioned in front of it. As most studies focusing on spatial imagery found, and in line with the perceptual anticipation theory, it is plausible to assume that primary visual areas were not active since specifically the spatial properties of the stimulus were recalled (Kosslyn et al., 2001; Kosslyn & Thompson, 2003; Mazard et al., 2004; Thompson et al., 2001).

Albeit several studies (Kosslyn et al., 1995; Kosslyn & Thompson, 2003; Pearson, 2019; Pearson & Kosslyn, 2015) have stressed the importance of early visual areas during imagery, subsequent work has led to contradictory findings (Lee et al., 2012) emphasizing instead the role of the HVC during imagery. Crucially, beyond the canonical activation analysis, a collection of studies using multivariate analyses (e.g., multivoxel pattern analysis and representational similarity analysis), has revealed that the areas of the HVC play a crucial role during imagery of scenes (Lee et al., 2012) and their locations (Boccia et al., 2015, 2017, 2021). With this in mind, to circumvent the potential difference between the imagery and the visual stimulus, in the DCM analysis, we specified condition‐specific connectivity models including only the scene‐selective and hippocampal regions, together with V1 in the visual perception.

## 5 CONCLUSION

Overall, our findings emphasize the neural mechanisms that distinguish imagery and perception of familiar scenes. Indeed, despite the common recruitment of areas in the ventral visual stream, qualitative and intrinsic differences in the connections among regions subserve these distinct processes. Also, the results of the present work highlight the role of RSC in visual perception and visual imagery of familiar scenes, revealing its central role in both conditions but with task‐dependent neural interactions with the other regions of the navigational network.

Our results are consistent with those of Lee et al. (2012) and Dijkstra et al. (2017) but go beyond them. To our knowledge, this is the first attempt to evaluate connectivity between broad brain areas processing scenes that intrinsically contain spatial information crucial to the aim of navigation. This distinctive feature of our task further underlined the RSC as a central hub for navigation. Indeed, under the same functional architecture, communications among regions subtend different causal influences depending on the task to be performed. Mapping the signal route from a common starting point to distinct regions depending on the image content during both perception and imagery would be a great future direction for research. Even though the current knowledge on brain activations during visual perception is fairly well known, many questions remain unanswered about the dynamic interaction between brain areas involved in both the perception and the imagery of the space around us.

## AUTHOR CONTRIBUTIONS

**Maria Giulia Tullo**: Conceptualization; methodology; formal analysis; software; visualization; writing—original draft. **Hannes Almgren**: Conceptualization; methodology; software; writing—review and editing. **Frederick Van der Steen**: Conceptualization; methodology; software; writing—review and editing. **Maddalena Boccia**: Investigation; resources; writing—review and editing. **Federica Bencivenga**: Methodology; software, writing—review and editing. **Gaspare Galati**: Methodology; writing—review and editing; supervision; project administration.

## CONFLICT OF INTEREST STATEMENT

The authors declare no conflict of interest.

## Data Availability

Data are available upon request to the corresponding author in compliance with the institutional ethics approval.
